# Spleen Stiffness Performance in the Noninvasive Assessment of Gastroesophageal Varices after Transjugular Intrahepatic Portosystemic Shunts

**DOI:** 10.1155/2021/5530004

**Published:** 2021-04-17

**Authors:** Huihui Zhou, Zhilin Zhang, Jun Zhang, Lin Sang, Lina Liu, Yong Lv, Xue Gong, Zhanxin Yin, Yuanyuan Sun, Guohong Han, Ming Yu

**Affiliations:** ^1^Department of Ultrasonic Medicine, Xijing Hospital, Fourth Military Medical University, Xi'an, Shaanxi, China 710032; ^2^Department of Liver Disease and Digestive Interventional Radiology, National Clinical Research Center for Digestive Diseases and Xijing Hospital of Digestive Diseases, Fourth Military Medical University, Xi'an, Shaanxi, China 710032

## Abstract

**Objectives:**

To investigate the performance of spleen stiffness (SS) by using two-dimensional shear-wave elastography (2D-SWE) for assessing the severity of gastroesophageal varices (GEVs) after transjugular intrahepatic portosystemic shunt (TIPS).

**Methods:**

102 eligible patients were categorized as in the post-TIPS short-term (*n* = 69) and long-term (*n* = 38) follow-up groups. The performance of SS by using 2D-SWE for evaluating the severity of GEVs was compared with liver stiffness (LS), spleen stiffness-to-liver stiffness ratio (SS/LS), liver stiffness spleen-diameter-to-platelet-ratio score (LSPS), portal hypertension (PH) risk score, platelet count-to-spleen diameter ratio (PSR), and varices risk score by using receiver operating characteristic (ROC) curve and DeLong test.

**Results:**

In the post-TIPS short-term follow-up group, area under the receiver operating characteristic curves (AUCs) of SS were 0.585 for mild (cutoff value = 30.3 kPa), 0.655 for moderate (cutoff value = 30.6 kPa), and 0.739 for severe (cutoff value = 31.9 kPa) GEVs, which were higher than other parameters for severe GEVs. AUCs of SS were lower than other parameters for mild and moderate GEVs, but no difference was found (*p* > 0.05). In the post-TIPS long-term follow-up group, AUCs of SS were 0.778 for mild (cutoff value = 28.9 kPa), 0.82 for moderate (cutoff value = 29.9 kPa), and 0.824 for severe (cutoff value = 37.7 kPa) GEVs, which were higher than other parameters except for severe GEVs. AUC of SS was lower than other parameters for severe GEVs, but no significant difference was found (*p* > 0.05).

**Conclusion:**

SS is an effective noninvasive tool to predict GEV severity during the post-TIPS follow-up.

## 1. Introduction

Transjugular intrahepatic portosystemic shunt (TIPS) is a feasible, safe, and effective treatment approach for the prevention and treatment for portal hypertensive complications [1, 2]. The 2-year mortality rate arising from gastroesophageal variceal (GEV) bleeding is 15-20% in patients with portal hypertension, and 1-year recurrence rate is 60% in patients without effective treatment [3, 4]. TIPS has been recommended and widely used in patients who failed endoscopic and pharmacological treatment for the prevention of GEV bleeding arising from portal hypertension by guidelines [5]. However, GEV rebleeding is one of the most complications after TIPS placement and can significantly influence patient life expectancy and quality of life [6]. Surveillance, prognosis, and management of gastroesophageal varices (GEVs) after TIPS are essential.

Upper gastrointestinal endoscopy screening is considered the reference standard for assessing the severity of GEVs. However, it does not meet the clinical needs because of their various potential complications and interobserver and intraobserver variability. Therefore, new noninvasive tools for evaluating the severity of GEVs in patients who underwent TIPS implantation have been an intense field of research.

Ultrasound elastography (i.e., acoustic radiation force impulse imaging (ARFI), transient elastography (TE), and point shear-wave elastography (pSWE)) and ultrasound elastography combine with biomarkers (i.e., liver stiffness spleen-diameter-to-platelet-ratio score (LSPS)) are widely used to predict the severity of GEVs in patients with portal hypertension [7–12]. However, among above studies, lots of participants with compensated advanced chronic liver disease did not undergo stent implantation; additionally, above elastography technologies obtained a lower success rate in patients with ascites, obesity, or narrow intercostal windows [13, 14]. Until now, only a few trials with small sample sizes were used to assess the hemodynamic change after TIPS placement [15–18].

Compared with TE, ARFI, and pSWE, two-dimensional shear-wave elastography (2D-SWE) is a new stiffness measurement technology with better diagnostic performance [13, 14], and then, its performance is not affected by ascites and obesity. It integrates B-mode map and 2D-SWE map in real time, so that nontarget structure can be validly avoided for obtaining more effective and reliable tissue stiffness measurement. However, the performance of 2D-SWE for predicting the severity of GEVs in patients who underwent TIPS implantation remains unclear.

We hypothesized that liver stiffness (LS) and/or spleen stiffness (SS) measured with 2D-SWE could effectively evaluate the severity of GEVs in patients who underwent TIPS placement, and that patients who underwent TIPS procedure could safely avoid upper gastrointestinal endoscopy screening during the follow-up.

## 2. Materials and Methods

### 2.1. Participants and Study Design

Between December 2016 and June 2019, a retrospective observational study was performed at Xijing Hospital (a tertiary university hospital in China). The study protocol was approved by the ethics committee of Xijing Hospital and in accordance with the ethical guideline of the 1975 Declaration of Helsinki and its later amendments. Informed consent was waived for the retrospective study.

All participants were enrolled in the research if they met the following criteria: (1) TIPS procedure was performed for GEVs arising from portal hypertension; (2) effective upper gastrointestinal endoscopy tests (range: 1-3 months in the post-TIPS [short-term follow-up group] and range: 4-12 months in the post-TIPS [long-term follow-up group], respectively); (3) effective abdominal US and 2D-SWE examinations (LS and SS) were performed on the same day; and (4) age 18-75 years. The exclusion criteria were as follows: (1) portal venous system thrombosis and cavernous transformation were confirmed by contrast-enhanced computed tomography (CECT) or US; (2) previous stent implantation and surgical treatment; (3) intrahepatic and extrahepatic malignancies, liver transplantation, and splenectomy; (4) 2D-SWE tests failed to obtain LS and SS values; and (5) upper gastrointestinal endoscopy examinations failed.

### 2.2. Abdominal US and 2D-SWE Examination

Abdominal US and 2D-SWE examinations were done by a single experienced sonographer, who had conducted more than 1000 abdominal US and 1000 2D-SWE examinations. All participants fasted for more than 8 hours before the examinations and were laid in the supine position with the right arm (LS measurement) or left arm (SS measurement) maximally lifted, fully showing a good visualization. The Aixplorer system (Aixplorer US system, SuperSonic Imagine; Aix-en-Provence, France) with a SC6-1 transducer (3.5 MHz convex transducer) was used.

In terms of abdominal US tests, the spleen length was measured as maximum spleen bipolar diameter in the longitudinal scan, and the thickness was measured as maximum diameter between splenic hilum and capsule in the transverse section scan. For above parameters, the final value for statistical analysis was the average of five repeated measurements. Subsequently, the LS and SS measure protocols were as follows [19, 20], which are also recommended by the latest EFSUMB guidelines [21]: (1) LS and SS values were measured through the right hepatic lobe and left intercostal windows, respectively; (2) region of interest (ROI) was located at a detecting depth at least 2 cm from the organ capsule and a well-visualized area that was free of vessels and bile ducts; (3) effective images included a ROI that filled at least 90% of the color map and stabilized for approximately 5 seconds were used for analysis, and the participants needed to hold their breath approximately 5 seconds during the examinations; (4) the diameter of the Q-box was 5-25 mm; and (5) for each participant, five and three consecutively effective 2D-SWE images were obtained in the liver and spleen, respectively. The mean value of the 2D-SWE expressed as kilopascals (kPa) was recorded.

### 2.3. TIPS Procedure

The TIPS procedure was performed as previously described and by the same experienced interventional team [22]. Briefly, an intrahepatic portosystemic shunt was created from the main portal vein (the caudal end of the shunt) to the hepatocaval junction (the cephalic end of the shunt) by using local anaesthesia at puncture point. All participants received TIPS with 8 mm polytetrafluoroethylene-covered shunts (Fluency^®^, Bard, Inc., Tempe, AZ, USA). During the TIPS procedure, the portosystemic pressure gradient (PPG) was measured preoperatively (baseline PPG) in order to evaluate the severity of portal hypertension, immediately after shunt implantation (immediate PPG) in order to evaluate the successfully performed of TIPS procedure. The PPG measurement was performed by using Mindray monitor (Mindray, Inc., Shenzhen, China).

### 2.4. Upper Gastrointestinal Endoscopy Examinations

After the US tests, upper gastrointestinal endoscopy examinations were done by experienced endoscopists (each with more than 8 years of experience), who were blinded to other examinations, including clinical information and US data. The results of GEV examination were recorded as LDRF classification reported by Li et al. [23], and the severity of GEVs was classified as mild, moderate, and severe according to the criteria used in National Clinical Research Center for Digestive Diseases and Xijing Hospital of Digestive Diseases (the high-level teaching hospital in China): mild, slightly linear expansions, red color signs negative (RC-: no blood vesicle, streak or cherry red signs, erosion, thrombus, and active bleeding); moderate, linear expansions with RC positive (RC+: blood vesicle, streak or cherry red signs, erosion, thrombus, and active bleeding) or serpentine expansions with RC-; severe, serpentine expansions with RC+ or nodular or neoplastic expansions with RC-/+.

### 2.5. Laboratory Data Collection

The laboratory data was collected from electronic medical records of patients within 1 week of 2D-SWE. The liver function, blood counts, and coagulation markers were collected. Noninvasive parameters were calculated as follows [11, 24–26]:

(1) $$\begin{array}{c} \text{SS}‐\text{to}‐\text{LS ratio}\left(\frac{\text{SS}}{\text{LS}}\right)=\frac{\text{spleen stiffness}}{\text{liver stiffness}},\\ \text{LS spleen}‐\text{diameter}‐\text{to}‐\text{platelet}‐\text{ratio score}\left(\text{LSPS}\right)=\frac{\left(\text{LS}\left[\text{by using either TE or }2\text{D}‐\text{SWE and given in kilopascals}\right]\times \text{spleen diameter}\left[\text{in centimeters}\right]\right)}{\text{platelet count ratio}\left(\times 10^{9}/\text{L}\right)},\\ \text{Platelet count}‐\text{to}‐\text{spleen diameter ratio }\left(\text{PSR}\right)=\frac{\text{platelet count }\left(\times 10^{9}/\text{L}\right)}{\text{spleen diameter }\left(\text{in millimeters}\right)},\\ \text{PH risk score}=\frac{-5.953+0.188\times \text{LS }\left(\text{by using either TE or }2\text{D}‐\text{SWE and given in kilopascals}\right)+1.583\times \text{sex }\left(1:\text{male};0:\text{female}\right)+26.705\times \text{spleen diameter }\left(\text{in millimeters}\right)}{\text{platelet count ratio }\left(\times 10^{9}/\text{L}\right)\;},\\ \text{Varices risk score}=-4.364+0.538\times \text{spleen diameter}-0.049\times \text{platelet count}-0.044\times \text{LS}+0.001\times \left(\text{LS}\times \text{platelet count}\right). \end{array}$$

### 2.6. Statistical Analysis

Continuous variables were shown as the medians with interquartile ranges (IQR). Comparisons between quantitative variables were performed by using the Wilcoxon matched-pair signed-rank test when appropriate. Categorical variables were shown as percentages and numbers when appropriate. The diagnostic performance of noninvasive parameters for evaluating the severity of GEVs (the presence of mild, moderate, and severe GEVs) was calculated using the receiver operating characteristic (ROC) curves and the areas under the receiver operating characteristic curves (AUCs). Differences between various AUCs of variables were compared by using DeLong tests. Sensitivity, specificity, and Youden index were calculated. The optimal cutoff values were determined as the sum of specificity and sensitivity. AUCs were provided with 95% confidence intervals (CIs).

The *p* values less than 0.05 were considered to indicate a significant difference. The data analyses were performed with the Statistical Analysis System 9.4 software (SAS Institute; Cary, NC) and GraphPad Prism version 6.0 (GraphPad Software, Inc., La Jolla, CA, USA).

## 3. Results

### 3.1. Baseline Characteristics of Participants

During the study period, 112 participants were screened, and 10 participants were excluded (Supplementary Figure 1). The reasons for exclusion were portal venous system thrombosis (*n* = 3), hepatocellular carcinoma (*n* = 1), failure to obtain LS and SS values (*n* = 4), splenectomy (*n* = 1), and a history of shunt placement (*n* = 1). Finally, 102 eligible participants were recruited in the clinical study and divided into the post-TIPS short-term (*n* = 69) and long-term (*n* = 38) follow-up groups (Supplementary Figure 1). The participants, including 51 (50%) males, had a mean age of 50.3 years (range: 18-75). The main etiologies were idiopathic portal hypertension (IPH, 43/102, 42.2%) and hepatitis B virus (HBV, 28/102, 27.5%). The baseline characteristics of participants were summarized in Table 1.

### 3.2. Diagnostic Performance of SS in Comparison with Other Noninvasive Parameters during the Post-TIPS Short-Term Follow-Up

In the post-TIPS short-term follow-up group, AUCs of SS (mild GEVs: AUC = 0.585, cutoff value = 30.3 kPa; moderate GEVs: AUC = 0.655, cutoff value = 30.6 kPa) were lower than other noninvasive parameters for mild and moderate GEVs (mild GEVs: LSPS, AUC = 0.609; PH risk score, AUC = 0.646; PSR, AUC = 0.594; varices risk score, AUC = 0.641. moderate GEVs: PH risk score, AUC = 0.688; varices risk score, AUC = 0.671) (Table 2), but no significant difference was found between AUCs of SS, LSPS, PH risk score, PSR, and varices risk score for predicting the present of mild GEVs and PH risk score and varices risk score for predicting the present of moderate GEVs (*p* > 0.05) (Supplementary Figure 3). SS indicated the highest AUC compared with all other noninvasive parameters for predicting the present of severe GEVs (AUC = 0.739) (Table 2, Supplementary Figure 3).

### 3.3. Diagnostic Performance of SS in Comparison with Other Noninvasive Parameters during the Post-TIPS Long-Term Follow-Up

In the post-TIPS long-term follow-up group, AUCs of SS were the highest compared with all other noninvasive parameters for predicting of mild (AUC = 0.778, cutoff value = 28.9 kPa) and moderate (AUC = 0.820, cutoff value = 29.9 kPa) (Table 3, Supplementary Figure 4). AUC of SS (AUC = 0.824, cutoff value = 37.7 kPa) was lower than other noninvasive parameters for severe GEVs (LSPS, AUC = 0.845; PH risk score, AUC = 0.832; PSR, AUC = 0.827; varices risk score, AUC = 0.907) (Table 3), but no significant difference was found between AUCs of SS, LSPS, PH risk score, PSR, and varices risk score for predicting the present of severe GEVs (*p* > 0.05) (Supplementary Figure 4).

## 4. Discussion

There are a few studies with small sample sizes that applied TE, ARFI, and pSWE focus on the evaluation of hemodynamic change after TIPS placement [15–18]. They all successfully demonstrated that SS showed the best diagnostic utility in reflecting the hemodynamic change after TIPS implantation compared with other noninvasive methods. However, there were some characteristics and limitations as follows: (1) the main objectives of early studies were not the severity of GEVs in the post-TIPS follow-up; (2) above elastography technology application and reliability are limited by obesity and ascites [13, 14]; (3) the small sample sizes and single-center characteristics had appreciable impact on the representativeness of the conclusion [15–18]; (4) just one of the former trials was conducted until 12 months after shunt placement [16]. Additionally, the cutoff values from TE, ARFI, and pSWE could not be directly applied to 2D-SWE examinations [27]. In our study, the diagnostic performance of SS, LS, SS/LS, LSPS, PSR, PH risk score, and varices risk score in evaluating the severity of GEVs was compared against upper gastrointestinal endoscopy screening in patients with portal hypertension during the post-TIPS follow-up. Our data indicated that the overall diagnostic performance of SS by using 2D-SWE was the best than other noninvasive parameters, such as LS, biomarkers, and LS combine with biomarkers, especially in the post-TIPS long-term follow-up.

In terms of the diagnostic performance of SS by using 2D-SWE for assessing the severity of GEVs in the post-TIPS short-term follow-up, the performance of SS from our study excepts severe GEVs (AUC = 0.739), because of AUCs < 0.70 for mild (AUC = 0.585) and moderate GEVs (AUC = 0.655). The possible reasons were as follows: (1) The hemodynamic changes would remarkably improve in the post-TIPS short-term follow-up [28], such as improvement in congestion of splanchnic, increase in central venous pressure and global blood flow, and increase in perfusion of splanchnic. Additionally, above hemodynamic changes would be stable approximately 1 month after TIPS placement [29]. However, the participants in our study are from different time points during the post-TIPS short-term follow-up. The instability of hemodynamic and difference in time point would influence the performance of SS. (2) Only 2.0% enrolled participants were in the mild GEVs and 10.0% in the moderate GEVs, which were much than less portion of participant in severe GEVs. The unbalanced samples further compromised the diagnostic utility of SS. The reason was that Xijing Hospital was a high-level teaching hospital in China, thus their patients were more likely to be in a severe stage. The possible approaches to improve diagnostic performance of SS during post-TIPS short-term follow-up are as follows: First, integrate multiple strategies for the time point. Second, SS by using 2D-SWE combine with biomarkers was performed. Third, large-scale of sample population and balanced data of different stage of GEVs is required.

During the post-TIPS long-term follow-up, SS showed the best diagnostic performance for predicting the presence of mild (AUC = 0.778) and moderate (AUC = 0.820) GEVs than other noninvasive parameters. For severe GEVs, compared with LSPS, PH risk score, PSR, and varices risk score, AUC of SS was lower (Table 3), but no significant difference was found between of them (Supplementary Figure 4). Previous studies demonstrated that in patients with the progression of portal hypertension, extrahepatic factors become the most important elements that associated with the further progression of portal hypertensive complications, such as congestion and perfusion, which was one of the most important reasons for increasing of SS at the decompensation stage of portal hypertension [16, 18, 29, 30]. Wang et al. suggested that blood volume of an 8-millimiter stent may importantly alleviate the blood volume of collateral circulation associated with portal hypertension; therefore, the slight change of hepatic perfusion is not enough to induce LS change [22]. Holland-Fischer et al. reported that the progression of fibrogenesis, angiogenesis, inflammation lymphoid hyperplasia, and serum markers did not improve in the post-TIPS follow-up [31], which were in accordance with the result from this study (Supplementary Table, Supplementary Figure 2). In conclusion, the performance of LS by using 2D-SWE, biomarkers, and LS combine with biomarkers was not satisfactory for predicting the severity of GEVs during the post-TIPS follow-up.

There were some certain limitations in our study. First, the single-center, small sample size characteristics may limit the representativeness of the conclusion. However, our cohort had better quality control than other similar studies: (1) the TIPS procedure was conducted by the same experienced team, and all patients received the same type of shunt; (2) the abdominal ultrasonographic examinations and 2D-SWE detections were carried out by the same experienced sonographer using the same type of ultrasonic machine. Second, in our studies, the upper gastrointestinal endoscopy screenings were performed by different endoscopic physicians, thus failing to avoid intraobserver variation. Third, as a rare disorder, idiopathic portal hypertension (IPH) is the main cause of GEVs in our study, and the possible reasons were as follows: (1) most of patients with viral hepatitis will prefer hepatology department, rather than Xijing hospital of digestive diseases; (2) Xijing hospital is a high-level teaching hospital in China; thus, its patients were more likely to be in a severe stage, and in addition, the GEVs in patients with IPH develop more severe and quick than those with viral hepatitis. Finally, IPH was the main etiology in our study due to the above reasons; the risk of selection bias is unavoidable. Fourth, we did not perform external validation in our study, which may limit representativeness of the conclusions. Additionally, the study was a retrospective design with variable follow-up intervals and protocols. Therefore, a prospective, external validation and large-scale of the diagnostic accuracy of SS by using 2D-SWE for predicting the severity of GEVs in the post-TIPS follow-up is required.

## 5. Conclusions

The study indicated that the AUC of SS was the highest than other parameters for assessing the present of severe GEVs and lower than other parameters for mild and moderate GEVs, but no difference was found during the post-TIPS short-term follow-up. The AUC of SS was the highest than other parameters for predicting the present of mild and moderate GEVs and lower than other parameters for evaluating the present of severe GEVs, but no difference was obvious. In addition, the SS measurement by using 2D-SWE was the easiest performed. All of these demonstrated a good potential of SS for assessing the severity of GEVs in patients underwent TIPS placement.

## Figures and Tables

**Table 1:** Baseline characteristics of patients (*n* = 102).

Characteristic	Values
Age (y)	50.3 (18.75)
No. of male^†^	51 (50%)
Etiology^†^	
ALD	3 (2.9)
CHF	3 (2.9)
CTPV	1 (1.0)
EHPVO	7 (6.9)
HBV	28 (27.5)
HCV	7 (6.9)
HBV and HCV	1 (1.0)
IPH	43 (42.2)
Miscellae	2 (2.0)
NAFLD	1 (1.0)
PBC	2 (2.0)
Unknown	3 (2.9)
Child-Pugh class^†^	
A (5-6)	37 (36.3)
B (7-9)	50 (49.0)
C (10-13)	15 (14.7)
Child-Pugh score^§^	7 (6,9)
MELD score^§^	8.6 (6.4, 11.2)
2D-SWE (kPa)^§^	
LS	12.7 (10.0, 18.3)
SS	39.8 (34.0, 46.4)
Conventional US^§^	
Spleen size (cm)	
Thickness	5.1 (4.5,5.8)
Diameters	16.5 (14.5,19.6)
Gastroesophageal endoscopy^†^	
Mild	2 (2.0)
Moderate	10 (9.8)
Severe	89 (87.3)
Complications pre-TIPS^†^	
Previous variceal bleeding	91 (81.3)
Acute bleeding	19 (17.0)
Massive ascites and previous variceal bleeding	10 (8.9)
Massive ascites, hydrothorax, and previous variceal bleeding	1 (0.9)
Massive hydrothorax and previous variceal bleeding	2 (1.8)
Previous treatment with endoscopic^†^	20 (17.9)
Previous treatment with beta-blockers^†^	85 (75.9)

ALD: autoimmune liver disease; CHF: congenital hepatic fibrosis; CTPV: cavernous transformation of the portal vein; EHPVO: extrahepatic portal vein obstruction; HBV: hepatitis B virus; HCV: hepatitis C virus; IPH: idiopathic portal hypertension; NAFLD: nonalcoholic fatty liver disease; PBC: primary biliary cirrhosis; MELD: model for end stage liver disease; 2D-SWE: two-dimensional shear-wave elastography; LS: liver stiffness; SS: spleen stiffness; TIPS: transjugular intrahepatic portosystemic shunt. ^†^Data are number of findings, with the percentage in parentheses. ^§^Data are medians, with the interquartile ranges in parentheses.

**Table 2:** Diagnostic performance of SS, LS, SS/LS, LSPS, PH risk score, PSR, and varices risk score for evaluating the severity of GEVs in the post-TIPS short-term follow-up.

Characteristic	AUC (%)	Cutoff value	Sensitivity (%)	Specificity (%)	Youden index
Mild GEVs					
SS	58.5	30.3	50.0	72.2	0.2
LS	54.7	13.6	35.9	83.3	0.2
SS/LS	55.0	3.6	25.9	100.0	0.3
LSPS	60.9	3.9	42.4	88.9	0.3
PH risk score	64.6	3.5	59.3	72.2	0.3
PSR	59.4	3.8	55.6	72.2	0.3
Varices risk score	64.1	2.3	42.4	89.9	0.3
Moderate GEVs					
SS	65.5	30.6	59.4	70.8	0.3
LS	53.6	10.4	76.7	44.2	0.2
SS/LS	59.8	4.0	32.1	93.8	0.3
LSPS	65.1	4.8	53.6	81.6	0.4
PH risk score	68.8	5.2	64.3	73.5	0.4
PSR	38.0	10.2	18.8	93.9	0.1
Varices risk score	67.1	3.2	46.4	95.9	0.4
Severe GEVs					
SS	73.9	31.9	83.3	67.6	0.5
LS	60.0	8.6	42.9	84.0	0.3
SS/LS	69.3	4.1	50.0	88.6	0.4
LSPS	57.3	2.5	83.3	46.5	0.3
PH risk score	62.6	5.0	83.3	59.2	0.4
PSR	36.7	26.0	16.7	100.0	0.1
Varices risk score	67.7	2.2	83.3	64.8	0.5

SS: spleen stiffness; LS: liver stiffness; SS/LS: spleen stiffness-to-liver stiffness ratio; LSPS: liver stiffness spleen-diameter-to-platelet-ratio score; PH risk score: portal hypertension risk score; PSR: platelet count-to-spleen diameter ratio; GEVs: gastroesophageal varices; TIPS: transjugular intrahepatic portosystemic shunt; AUC: areas under the receiver operating characteristic curve. AUC of SS was statistically compared with AUC of LS, SS/LS, LSPS, PH risk score, PSR, and varices risk score, respectively, in the same GEVs stage (*p* > 0.05).

**Table 3:** Diagnostic performance of SS, LS, SS/LS, LSPS, PH risk score, PSR, and varices risk score for evaluating the severity of GEVs in the post-TIPS long-term follow-up.

Characteristic	AUC (%)	Cutoff value	Sensitivity (%)	Specificity (%)	Youden index
Mild GEVs					
SS	77.8	28.9	68.2	88.9	0.6
LS	70.9	7.9	91.0	50.0	0.4
SS/LS	56.9	3.5	76.2	55.6	0.3
LSPS	72.0	1.7	76.2	66.7	0.4
PH risk score	75.1	-0.1	95.2	55.6	0.5
PSR	65.7	6.0	77.3	66.7	0.4
Varices risk score	73.0	-0.7	76.2	66.7	0.4
Moderate GEVs					
SS	82.0	29.9	83.3	78.9	0.6
LS	62.9	12.8	50.0	75.0	0.3
SS/LS	57.7	3.2	72.7	63.2	0.4
LSPS	68.4	3.1	63.6	78.9	0.4
PH risk score	68.9	5.0	63.6	78.9	0.4
PSR	64.0	4.6	83.3	52.6	0.4
Varices risk score	69.4	2.1	63.6	84.2	0.5
Severe GEVs					
SS	82.4	37.7	71.4	95.8	0.7
LS	55.4	13.2	42.9	76.0	0.2
SS/LS	62.1	3.2	85.7	60.9	0.5
LSPS	84.5	3.3	85.7	82.6	0.7
PH risk score	83.2	6.8	85.7	87.0	0.7
PSR	82.7	2.6	71.4	91.7	0.6
Varices risk score	90.7	2.1	100.0	87.0	0.9

SS: spleen stiffness; LS: liver stiffness; SS/LS: spleen stiffness-to-liver stiffness ratio; LSPS: liver stiffness spleen-diameter-to-platelet-ratio score; PH risk score: portal hypertension risk score; PSR: platelet count-to-spleen diameter ratio; GEVs: gastroesophageal varices; TIPS: transjugular intrahepatic portosystemic shunt; AUC: areas under the receiver operating characteristic curve. AUC of SS was statistically compared with AUC of LS, SS/LS, LSPS, PH risk score, PSR, and varices risk score, respectively, in the same GEVs stage (*p* > 0.05).

## Data Availability

The data used and analyzed during the current study are available from the corresponding author on reasonable request.

## Abbreviations

SS: Spleen stiffness
GEVs: Gastroesophageal varices
TIPS: Transjugular intrahepatic portosystemic shunt
2D-SWE: Two-dimensional shear-wave elastography
PH: Portal hypertension
ROC: Receiver operating characteristic
AUCs: Area under the receiver operating characteristic curves
PPG: Portosystemic pressure gradient
US: Ultrasound
LS: Liver stiffness
ARFI: Acoustic radiation force impulse imaging
TE: Transient elastography
pSWE: Point shear-wave elastography
IVC: Inferior vena cava
ROI: Region of interest
kPa: Kilopascals
SS/LS: Spleen stiffness-to-liver stiffness
LSPS: Liver stiffness spleen-diameter-to-platelet-ratio score
PSR: Platelet count-to-spleen diameter ratio
IQR: Interquartile ranges
CIs: Confidence intervals
IPH: Idiopathic portal hypertension
HBV: Hepatitis B virus
OR: Odds ratio
CVP: Central venous pressure.

## Supplementary Materials

Supplementary 1 Supplementary Figure 1: flowchart of the study design. TIPS: transjugular intrahepatic portosystemic shunt; LS: liver stiffness; SS: spleen stiffness.

Supplementary 2 Supplementary Figure 2: the B-mode (bottom) and 2D-SWE (top) liver and spleen images of patients were displayed side by side between pre-TIPS and in the post-TIPS short-term (a–d) and long-term (e–h) follow-up groups. (a, b, e, f) LS measurement; (c, d, g, h) SS measurement.

Supplementary 3 Supplementary Figure 3: comparison of ROC curves between SS, LS, SS/LS, LSPS, PSR, PH risk score, and varices risk score for predicting the severity of GEVs in the post-TIPS short-term follow-up. (A, C, E) SS versus LS, SS/LS, and PSR for mild, moderate, and severe GEVs, respectively (*p* > 0.05). (B, D, F) SS versus LSPS, PH risk score, and varices risk score for mild, moderate, and severe GEVs, respectively (*p* > 0.05).

Supplementary 4 Supplementary Figure 4: comparison of ROC curves between SS, LS, SS/LS, LSPS, PSR, PH risk score, and varices risk score for predicting the severity of GEVs in the post-TIPS long-term follow-up. (A, C, E) SS versus LS, SS/LS, and PSR for mild, moderate, and severe GEVs, respectively (*p* > 0.05). (B, D, F) SS versus LSPS, PH risk score, and varices risk score for mild, moderate, and severe GEVs, respectively (*p* > 0.05).

Supplementary 5 Supplementary Table: comparison of parameters between pre-TIPS and post-TIPS.
