# Supplementary material for: Spleen Stiffness Performance in the Noninvasive Assessment of Gastroesophageal Varices after Transjugular Intrahepatic Portosystemic Shunts
Source: Biomed Res Int. 2021 Apr 17;2021:5530004. doi: 10.1155/2021/5530004 (PMC8077793; doi:10.1155/2021/5530004)
Supplement: Supplementary 5 — Supplementary Table: comparison of parameters between pre-TIPS and post-TIPS. [file 5530004.f5.docx]

**Supplementary table.** Comparison of parameters between post-TIPS and pre-TIPS.

TIPS: transjugular intrahepatic portosystemic shunt; 2D-SWE: two-dimensional shear-wave elastography.

‡ data are means ± standard deviations.
